# A sudden but prolonged collective trauma: The Ukrainian experience

**DOI:** 10.1371/journal.pmen.0000056

**Published:** 2024-06-10

**Authors:** Nataliia Frolova, Roxane Cohen Silver

**Affiliations:** 1 Department of Psychology and Social Work, Oles Honchar Dnipro National University, Dnipro, Ukraine; 2 Department of Psychological Science, University of California, Irvine, Irvine, California, United States of America; 3 Department of Medicine and Program in Public Health, University of California, Irvine, Irvine, California, United States of America; PLOS: Public Library of Science, UNITED KINGDOM

On February 24, 2022, the Russian Army invaded Ukraine, almost without warning. From the perspectives of a Ukrainian psychologist who lived through the invasion and another who has studied coping with traumatic experiences for four decades, we seek to offer an analysis of the mental health impact of the war on the residents of Ukraine. The main focus of our attention is on the civilian population who did not leave the country or returned after some time spent abroad. These people, from our point of view, have the greatest need for an effective support system for coping with their traumatic experiences.

## War context

The invasion of Ukraine has resulted in people being forced to flee their homes or trying to survive in the extreme conditions of conflict. They are in dire need not only of humanitarian aid but also psychological assistance. According to the Ukrainian Ministry of Foreign Affairs, as of June 21, 2023, 8,177,000 Ukrainian citizens are living abroad, which represents about 20% of the population of Ukraine at the start of the war. In addition to migration abroad, Ukraine has also faced internal migration. According to the Ministry of Social Policy, under 4,900,000 Ukrainians have the status of internally displaced people [1].

Ukraine is in a hybrid war, which is defined by North Atlantic Treaty Organization (NATO) as “an interplay or fusion of conventional as well as unconventional instruments of power and tools of subversion”. Some hybrid methods of warfare have been used in the past, but the general speed, scale and intensity of their emergence due to technological progress and global interconnectivity, arguably make it possible to regard them as a qualitatively new experience [2]. Hybrid military strategies, propaganda, and disinformation aimed at creating panic, confusion, fear, and distrust are the face of modern warfare, which threatens, injures and takes Ukrainian civilians’ lives. This hybrid character of the war is complicated by the fact that the warring countries have the same language group (which makes it possible to perceive news from both sides), religious beliefs, and a large number of friends and relatives on the opposite side.

After over two years of conflict, the war has become a major polytraumatic event. As civilian attacks have been carried out throughout the country, there is no place in Ukraine where civilians can feel safe.

Although the war started suddenly for the majority of the civilian population, today it has a long-term character and has led to chronic traumatization [3], which can exist on at least two levels at once—individual and collective—and affects every Ukrainian living in, as well as out of, the country. This traumatization has touched human, social, democratic, material, economic and environmental aspects of people’s life [4].

## The Ukrainian traumatic experience

One must consider the Ukrainian experience as a process, with its own specificity and characteristics. The war has continued unabated for over two years; the trauma has thus been chronic. Moreover, current events are superimposed on an 8-year period of anti-terrorist operations that preceded it (2014–2022), as well as the COVID-19 pandemic, which had been ongoing for two years before the invasion.

Specifically, Ukrainian people are grappling with intense, direct exposure to cascading events [5] such as random civilian bombings and terror attacks, personal and economic losses, and social isolation from relatives and friends. In addition, the Ukrainian population has been repeatedly traumatized by transmission of the Russian invasion via both traditional and social media. This way of receiving information is one of the most traumatic, due to the intense and rapid dissemination of negative stories and graphic visual images, as well as being easily accessed. The parallel experiences of both individual and collective traumatization have impacted individual mental, physical, and public health [6].

## Three periods of the Russian-Ukrainian war 2022

When discussing the psychological impact of the war on the Ukrainian people, we divide the war into 3 periods of six months each to address the mental health effects and identify the features of the population’s experiences during each period.

### 1. February 24, 2022-August 2022

The initial reaction of Ukrainians on February 24 2022 was likely to be one of shock. Cities in different parts of the country came under fire and by the middle of the day a panic seized almost everyone—there were long queues at supermarkets, pharmacies and gas stations. But at the same time, many went to donate blood; the City Territorial Defenses began building fortifications, with long lines of volunteers at the military commissaries. As the Ukrainian Army was not a reserve military force at that time, the decision to defend the country was taken by the civilians working as computer specialists, bank employees, office managers, etc. By October 16, 2022, more than 100,000 Ukrainian civilians had joined the military forces [7].

This period was characterized by a very high level of anxiety, fear for adults’ and children’s lives, and overwhelming uncertainty. During the first weeks of the war, any movement across the country was accompanied by large crowds of people and outbursts of aggression and panic.

During this period of time, psychological assistance was in relatively low demand as people focused on their basic survival needs. Many ran out into the street without any belongings, stopped transportation, and left the cities that were bombed. Meeting basic needs (for food, clothing, shelter, etc.) was dominant. Nevertheless, psychologists were on duty in all shelters and humanitarian aid centers and provided psychological support to those who requested it.

During this time, volunteering in Ukraine became a form of coping that allowed civilians to get involved on behalf of the war effort–and this continued as discussed below. Furthermore, a landmark moment of this period was increased patriotism, which has continued to rise.

### 2. August 2022 -February 2023

The second period was characterized as a plateau where actions and plans were stabilized for the majority of Ukrainian civilians. The number of Ukrainians leaving the country decreased by 9 times during that period—about 500,000 compared to 4,600,000 Ukrainians who left from February 2022 to August 2022 [8]. Most of the temporarily displaced people within the country began to adapt to their new location.

At the same time, the necessity of psychological help was not still sufficiently urgent and most of the requests came from people who worked in the “human-human” system (social workers, volunteers) and appeared to be experiencing emotional burnout.

### 3. February 2023-August 2023

The Ministry of Social Policy reported that as of June 9, 2023, 4,871,807 people were registered as internally displaced in Ukraine, of whom 60% were women and 40% were men. It should be emphasized that the full-scale invasion forced 700 people aged 100 and older to leave their homes (1). Representative surveys conducted at that time revealed the key emotions of Ukrainians when they thought about the country’s future were hope (59%), anxiety (42%), and optimism (33%). Compared to December 2022, negative emotions such as anxiety (+18%), fear (+14%), and confusion (+10%) were more frequent among the population, while fewer citizens were optimistic about the future of Ukraine (-7%) [9].

The third period is characterized as having the highest number of requests for psychological help, which doubled compared to 2022 [10], against the background of complaints about the worsening of physical health. Individuals reported symptoms of insomnia, apathy, increased anxiety, aggressive behavior, and guilt for reactions toward loved ones.

Simultaneously, in 2023 Ukraine was ranked second out of 142 countries in the World Giving Index, an annual report published by the Charities Aid Foundation that ranks countries across the world according to how charitable) their residents are. Today 78% of Ukrainians help strangers, 70% donate money and 37% are official volunteers [11]. It is likely that Ukrainians continue to volunteer as a strategy that allows them to cope with their traumatic experience.

## A call to action

Today the Ukrainian experience is chronic and remains brutal and unpredictable. Its duration gives rise to a lack of physical and emotional security anywhere in the country. From a mental health perspective, the primary goal now is to help civilians understand their emotions and thoughts in the context of the war so that they are ready to receive psychological support.

In the midst of ongoing challenges, Ukrainians must cultivate their resilience and values (the highest of which is the appreciation of human life) and learn to navigate the new reality. Ukrainians know all too well what this means and involves. Aside from ensuring that Ukrainians receive the mental health support that they need, we hope that their situation and resilience can serve as a model to help us to gain understanding of trauma that can be expanded on a global scale for all challenged societies.
