# Correction: A sudden but prolonged collective trauma: The Ukrainian experience

**DOI:** 10.1371/journal.pmen.0000658

**Published:** 2026-07-10

**Authors:** Nataliia Frolova, Roxane Cohen Silver

There is an error in affiliation 1 for author Nataliia Frolova. The correct affiliation 1 is: Faculty of Psychology and Special Education, Oles Honchar Dnipro National University, Dnipro, Ukraine.
